# Therapeutic vaccine targeting Epstein-Barr virus latent protein, LMP1, suppresses LMP1-expressing tumor growth and metastasis in vivo

**DOI:** 10.1186/s12885-016-3027-1

**Published:** 2017-01-05

**Authors:** Mei-Chun Lin, Yong-Chong Lin, Syue-Ting Chen, Tai-Horng Young, Pei-Jen Lou

**Affiliations:** 1 Department of Otolaryngology, National Taiwan University Hospital, Hsin-Chu Branch, No. 25, Lane 442, Sec. 1, Jingguo Road, Hsinchu City, 300 Taiwan; 2 Graduate Institute of Anatomy and Cell Biology, National Taiwan University College of Medicine, No. 1, Sec. 1, Jen-Ai Road, Taipei, 100 Taiwan; 3 Institute of Biomedical Engineering, College of Medicine and College of Engineering, National Taiwan University, No. 1, Sec. 1, Jen-Ai Road, Taipei, 100 Taiwan; 4 Department of Otolaryngology, National Taiwan University Hospital and College of Medicine, No. 7, Chung-Shan South Road, Taipei, 100 Taiwan

**Keywords:** Epstein-Barr virus (EBV), Latent membrane protein 1 (LMP1), Nasopharyngeal carcinoma (NPC), DNA vaccine, Cytotoxic T lymphocyte (CTL)

## Abstract

**Background:**

In endemic area, nasopharyngeal carcinoma (NPC) tumor cells harbor EBV latent infection and expresses viral antigens such as EBNA1, LMP1 and LMP2. In this study, we established a NPC-mimicry animal model and assessed the therapeutic potential of LMP1 vaccine.

**Methods:**

Animal models were established by injection of LMP1-expressing TC-1 cells in C57BL6/J mice subcutaneously or through tail veins. pcDNA3.1 empty vector or *LMP1*/pcDNA3.1 vaccine was delivered by a helium-driven gene gun. Effectiveness of vaccine was evaluated by measuring the tumor size and numbers of metastatic lung nodules. Circulating cytokines were evaluated by ELISArray. Populations of activated cytotoxic T lymphocytes (CTLs) and LMP1-specific T lymphocytes were evaluated by flow cytometry with CD8/CD107a double staining and interferon-γ ELISPOT assay, respectively.

**Results:**

LMP1 vaccine significantly suppressed tumor growth (*n* = 3) and metastasis (*n* = 4) in vivo. When vaccinated before tumor challenge, all mice in vaccine group were tumor-free, whereas all mice in the control group developed tumors within 2 weeks after tumor challenge (*n* = 10). Cytokine ELISArray revealed elevation of a panel of proinflammatory cytokines in mice receiving LMP1 vaccine. Flow cytometry and interferon-γ ELISPOT assay revealed that LMP1 vaccine induced larger populations of activated CTLs and LMP1-specific T lymphocytes.

**Conclusions:**

This pre-clinical study provides a promising result that LMP1 vaccine suppresses LMP1-expressing tumor growth and metastasis in vivo.

## Background

Epstein-Barr virus (EBV), a member of the herpesvirus family, was the first virus identified to correlate with human malignancies such as Hodgkin’s disease (HD), Burkitt’s lymphoma, nasopharyngeal carcinoma (NPC) and gastric carcinoma [1]. Different from other type of cancers, virus-associated malignancies express foreign epitopes by major histocompatibility complex (MHC) molecules, which can be recognized by cytotoxic T lymphocytes (CTLs). Therefore, immunogenicity of latent viral proteins and strategies to activate antigen-specific CTLs has become the focus of immunotherapies against virus-associated malignancies in recent decades [2].

NPC arises from epithelia of the nasopharynx and almost all tumors in the endemic area including Southern and South-Eastern Asia, are associated with EBV infection [3]. The mortality rate of NPC has been decreasing in the past two decades owing to advances to multidisciplinary treatment including radiation and combination chemotherapy. However, the failure to treat locally-aggressive nasopharyngeal carcinoma is not uncommon and distant metastases remains the main cause of death [4]. The strong association of NPC with EBV makes viral antigen-targeted immunotherapy an attractive treatment modality [5]. Adoptive transfer of autologous EBV-specific CTL has proven effective in metastatic or locally recurrent NPC patients [6]. Meanwhile, efficacies of DNA vaccines carrying various combination of EBV antigens are also under evaluation [7–9].

NPC, as well as HD, represents the classic type II latent infection by the expression of EBV nuclear antigen 1 (EBNA1), latent membrane proteins (LMP) 1 and 2 [10]. EBNA1 evades CTL recognition by blocking MHC complex I epitopes presentation [11, 12]. However, LMP1 and LMP2 are relatively immunogenic and have been proposed as therapeutic targets [13, 14]. In this study, we establish a NPC-mimicry animal model and demonstrate that *LMP1* encoding DNA vaccine is effective in tumor suppression and prevention. In addition, we also show that LMP1 vaccine successfully induces elevation of proinflammatory cytokines and activates LMP1-specific CTLs. Taken together, these results support the implication of LMP1-based DNA vaccine in clinical use in the future.

## Methods

### Cell culture

Mus musculus lung tumor cell line, TC-1, was kindly provided by Dr. Wen-Fang Cheng (National Taiwan University Hospital) [15] and cultured in RPMI-1640 (Invitrogen) supplemented with 10% fetal bovine serum (FBS, PAA laboratories), 50 U/mL of penicillin/streptomycin, 2 mM of L-glutamine, 1 mM of sodium pyruvate, 2 mM of non-essential amino acids and 0.4 mg/mL of G418 (Sigma-Aldrich) at 37 °C with 5% CO_2_.

### Plasmid construction

Primary NPC tissues were obtained with inform consents from patients (IRB number: 201304078RIND). Total RNA was extracted from primary NPC tissues with TRIzol^®^ Reagent (Invitrogen) according to the manufacturer’s protocol. Reverse transcription reaction was performed using the Superscript III First-Strand cDNA Synthesis Kit (Invitrogen) with 2 μg of total RNA. *LMP1* was obtained by real-time RT-PCR with the following primers, 5′-ggatccgccatggaacacgaccttgagaggggcccac-3′ and 5′-gcggccgctcatagtagcttagctgaactgggccgtg-3′. *LMP1* was subcloned into pLenti6/V5-DEST with BamHI and NotI as restriction sites. Lentivirus expression system was carried out according to the manufacturer’s protocol (Invitrogen). Stable Mock (pLenti6/V5-DEST) or LMP1 (*LMP1*/pLenti6/V5-DEST) TC-1 clones were selected with 3 μg/mL blasticidin (Invitrogen). For generation of DNA vaccine, the CRT/pcDNA3.1 vector was a gift from Dr. Wen-Fang Cheng [15]. *LMP1* was cloned from *LMP1*/pLenti6/V5-DEST with the following primers, 5′-gaattcatggaacacgaccttgagag-3′, 5′-aagcttttagtcatagtagcttagct-3′ and subcloned into CRT/pcDNA3.1 vectors using the restriction site of EcoRI and HindIII. All constructs were verified by DNA sequencing.

### Western blot analysis

Cell pellets were homogenized in lysis buffer containing 1% Triton X-100, 20 mM Tris-HCl, pH 8.0, 160 mM NaCl, 1 mM CaCl_2_, and 1 mM phenylmethysulfonyl fluoride. Insoluble material was removed by centrifugation at 14,000 rpm for 15 min. Cell lysates were boiled at 95 °C for 5 min in SDS-buffer containing mercaptoethanol. Twenty micrograms of the protein sample were separated on an 8% SDS-PAGE and transferred to the nitrocellulose membrane (GE Healthcare). LMP1 proteins were detected with mouse anti-LMP1 monoclonal antibody (Abcam). β-actin was an internal control and detected with rabbit anti-β-actin polyclonal antibody (GeneTex).

### Animals

All animal experiments were approved by Institutional Animal Care and Use Committee of National Taiwan University College of Medicine, Taipei, Taiwan. C57BL6/J mice (8 weeks) were obtained from the National Laboratory Animal Center, Taipei, Taiwan.

### Immunohistochemistry

Xenografts were paraffin-embedded and incubated with mouse anti-LMP1 monoclonal antibodies (1:400, Abcam) diluted with 5% bovine serum albumin/PBS for 12 h at 4 °C. After rinsing twice with PBS, Super Sensitive Link-Label Immunohistochemistry Detection System (BioGenex) was used and the specific immunostaining was visualized with 3,3-diaminobenzidine liquid substrate system (Sigma-Aldrich). All sections were counterstained with hematoxylin.

### In vivo tumorigenesis in C57BL6/J mice

For subcutaneous xenografts and experimental metastasis, C57BL6/J mice were injected with LMP1-expressing TC-1 cells, subcutaneously or through tail veins, respectively. On the 7th day after tumor inoculation, vaccination with empty vector or *LMP1*/pcDNA3.1 was given. Combination with intraperitoneal injection of cisplatin (10 mg/kg) was added in experimental metastasis model. For tumor prevention, mice were vaccinated 3 weeks before tumor inoculation. Subcutaneous tumor growth was measured twice per week and tumor volumes were estimated by modified ellipsoid formula: (Length × Width^2^)/2.

### Immunization of C57BL6/J mice

Empty vector or *LMP1*/pcDNA3.1 plasmid were coated on to gold particles by incubating with spermidine (Sigma-Aldrich) on ice for 10 min. The DNA coated gold particles were delivered to the shaved abdominal area of the mice through a helium-driven gene gun (BioRad Laboratories Inc.)

### Flow cytometry

For detection of activated CTLs, 1 × 10^6^ mouse splenocytes were incubated with FITC-conjugated goat anti-mouse CD107a antibodies (1:100, eBiosceince), and PE-conjugated goat anti-mouse CD8 antibodies (1:100, eBioscience) on ice for 30 min. Immunofluorescence was detected by flow cytometry analysis (Becton-Dickinson).

### Cytokine ELISArray

Mouse cytokine Multi-Analyte ELISArray was carried out according to manufacturer’s protocol (Qiagen). Briefly, Millipore 96-well plates (R & D system) were incubated with mouse sera (1:4) for two hours in room temperature. Detection antibodies were then added and incubated for two hours in room temperature. Avidin-HRP was added for another 1 h under room temperature. Development solution were then added and ELISA reader was used to detect O.D. at 450 nm.

### Interferon (INF)-γ ELISPOT assay

Millipore opaque 96-well plates (R & D system) were incubated with RPMI-1640 for 20 min. Splenocytes were harvested from both control or *LMP1*/pcDNA3.1 vaccinated mice and re-suspended with RPMI-1640 supplemented with 10% fetal bovine serum, 50 μM β-mercaptoethanol, 100 U/ml penicillin and 100 μg/ml streptomycin. The harvested splenocytes were counted to the final concentration of 1 × 10^6^ cell/well and were incubated in 37 °C at 5% CO_2_ for 24 h with or without PepMix EBV-LMP1 peptides (1 μg/ml, JPT Peptide Technologies). Each well was triplicated. After incubation, biotinylated anti-mouse IFN-γ antibodies were added and incubated at 4 °C for 18 h. Then, Streptavidin-AP was added to each well and incubated at room temperature for 2 h. Finally, BCIP/NBT Chromogen were used for color development. The spot forming cells (SFCs) were counted by automatically by the ImmunoSpot Analyzer (Cellular Technology, Ltd).

### Statistical analysis

Student’s *t*-test was used for statistical analyses. Data are presented as mean ± S.D. and *P* < 0.05 is considered statistically significant.

## Results

### LMP1 enhances tumor growth of TC-1 cells in vivo

**Fig. 1:**
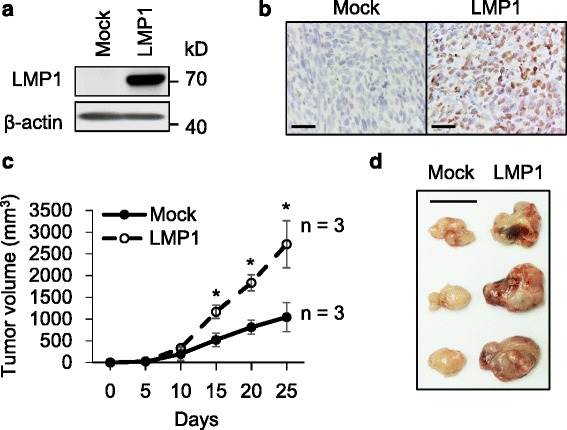
LMP1 enhances tumor growth of TC-1 cells in vivo. **a** Western blot analysis of LMP1 expression in TC-1 cells. TC-1 cells were transfected with either pLenti6/V5-DEST (Mock) or *LMP1*/pLenti6/V5-DEST (LMP1) plasmid. Stable Mock or LMP1-expressing cells were selected with blasticidin (3 μg/ml) for 2 weeks before further experiments. β-actin was an internal control. **b** Immunohistochemistry of LMP1. Paraffin embedded Mock or LMP1 xenografts were stained with anti-LMP1 antibody. Scale bar, 50 μm. **c** Effects of LMP1 on tumor growth in vivo. C57BL6/J mice were subcutaneously injected with 1 × 10^5^ of ether Mock or LMP1-expressing TC-1 cells (*n* = 3) and tumor sizes were measured every 5 days. *, *P* < 0.05. **d** Images of Mock and LMP1 xenografts. Scale bar, 1 cm

To establish a LMP1-expressing tumor model which mimics NPC, TC-1 cells were stably transfected with either empty vector (Mock) or *LMP1*/pLenti6/V5-DEST (LMP1). The expression of LMP1 was confirmed by Western blot analysis (Fig. 1a). Next, we investigated effects of LMP1 on tumor growth by subcutaneous injection of Mock or LMP1-expressing TC-1 cells on C57BL6/J mice. Expressions of LMP1 in Mock and LMP1 xenografts were analyzed by immunohistochemistry (Fig. 1b). Results showed that LMP1 expression significantly increased tumor growth in vivo (Fig. 1c and d). Consistent with the established oncogenic nature of LMP1 [16, 17], these results show that ectopic LMP1 expression promotes TC-1 tumor growth in vivo.

### Vaccination of *LMP1*/pcDNA3.1 suppresses LMP1-expressing tumor growth in vivo

**Fig. 2:**
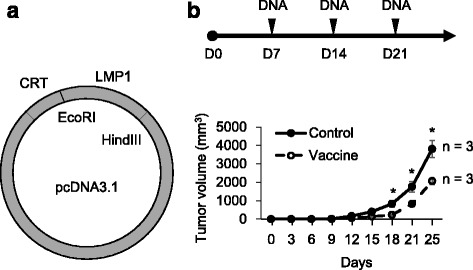
Vaccination of *LMP1*/pcDNA3.1 suppresses LMP1-expressing tumor growth in vivo. **a** *LMP1*/pcDNA3.1 vaccine construct. Restriction sites are as indicated. CRT, calreticulin. **b** Effect of vaccination on LMP1-expressing tumor growth in vivo. *Upper panel*, schedule of tumor injection and vaccination. *Lower panel*, tumor growth curves. *, *P* < 0.05. C57BL/6 J mice were subcutaneously injected with 1 × 10^5^ LMP1-expressing TC-l cells (D0). Vaccination with either empty pcDNA3.1 (control) or *LMP1*/pcDNA3.1 (vaccine) (*n* = 3) were given weekly on the 7th day after tumor injection for 3 consecutive weeks. Tumor volumes were measured every 3 days and the mice were sacrificed on the fourth week

After establishment of the LMP1-expressing tumor model, we designed a calreticulin conjugated DNA vaccine that encoded full-length *LMP1* (*LMP1*/pcDNA3.1, Fig. 2a). Next, we evaluated efficacies of LMP1 vaccine treatment on tumor growth. C57BL6/J mice were subcutaneously injected with LMP1-expressing TC-1 cells and then vaccinated weekly with empty pcDNA3.1 (control) or *LMP1*/pcDNA3.1 (vaccine) for three consecutive weeks (Fig. 2b, upper panel). Results showed that vaccination of *LMP1*/pcDNA3.1 significantly suppressed LMP1-expressing tumor growth as compared with controls (Fig. 2b, lower panel). These results suggest that LMP1 vaccine is effective in suppression of LMP1-expressing tumor growth in vivo.

### Vaccination of *LMP1*/pcDNA3.1 suppresses LMP1-expressing tumor metastasis in vivo

**Fig. 3:**
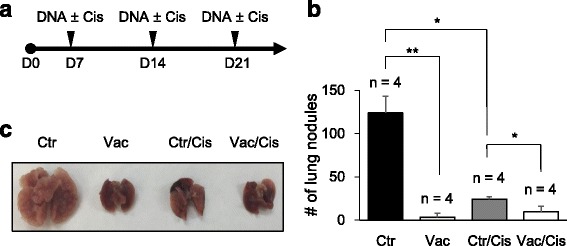
Vaccination of *LMP1*/pcDNA3.1 suppresses LMP1-expressing tumor metastasis in vivo. **a** Schedule of tumor injection and vaccination with or without cisplatin. C57BL/6 J mice were intravenously injected with 1 × 10^5^ LMP1-expressing TC-l cells (D0). On the 7th day after tumor injection, 2 μg of either empty vector (control) or *LMP1*/pcDNA3.1 (vaccine) was delivered to abdominal area weekly for 3 consecutive weeks. For evaluation of combination therapy, cisplatin (10 mg/kg) was given intraperitoneally at the same time of vaccination. Mice were sacrificed on the fourth week after tumor injection. **b** Number of lung nodules in each group with different treatments (*n* = 4). *, *P* < 0.05. **, *P* < 0.01. **c** Representative images of lungs

To evaluate the effects of LMP1 vaccine on tumor metastasis, C57BL6/J mice were injected with LMP1-expressing TC-1 cells through tail veins and then vaccinated with or without cisplatin weekly for 3 consecutive weeks (Fig. 3a, upper panel). Results showed that *LMP1*/pcDNA3.1 vaccination significantly suppressed tumor metastasis as compared with controls (Fig. 3b, first and second column). Interestingly, although cisplatin alone also significantly decreased the number of lung nodules as compared with controls, combination of cisplatin with LMP1 vaccine was not significantly more effective than LMP1 vaccine alone (Fig. 3b, second and fourth column). These results indicate that *LMP1*/pcDNA3.1 vaccination is effective in suppression of LMP1-expressing tumor metastasis in vivo.

### Vaccination of *LMP1*/pcDNA3.1 prevents LMP1-expressing tumor development in vivo

**Fig. 4:**
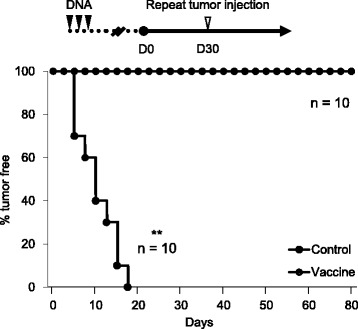
Vaccination of *LMP1*/pcDNA3.1 prevents LMP1-expressing tumor development in vivo. *Upper panel*, schedule of vaccination and tumor injection. *Lower panel*, percentage (%) of mice remained without tumor. C57BL/6 J mice were vaccinated weekly with either empty vector (control) or *LMP1*/pcDNA3.1 (vaccine) (*n* = 10) for 3 weeks. Subcutaneous injection of 1 × 10^5^ LMP1-expressing TC-1 cells was given 3 weeks after the last dose of vaccination (D0). Tumor volumes were measured every 3 days. Re-challenge of 1 × 10^6^ LMP1-expressing TC-1 cells was given to vaccine group on the 30th day. No tumor growth was observed until the 80th day. **, *P* < 0.01

To evaluate effects of LMP1 vaccine on disease prevention, three consecutive doses of vaccination were given before subcutaneous injection of LMP1-expressing TC-1 cells (Fig. 4, upper panel). Results showed that all mice in the control group developed tumor before the end of the third week. In contrast, no tumor growth was observed in the LMP1 vaccinated group even after repeated tumor injection on the 30th day (Fig. 4, lower panel). These results suggest that *LMP1*/pcDNA3.1 vaccine is effective in prevention of LMP1-expressing tumor growth in vivo.

### *LMP1*/pcDNA3.1 vaccination increases activated CTLs and induces proinflammatory cytokines in vivo

**Fig. 5:**
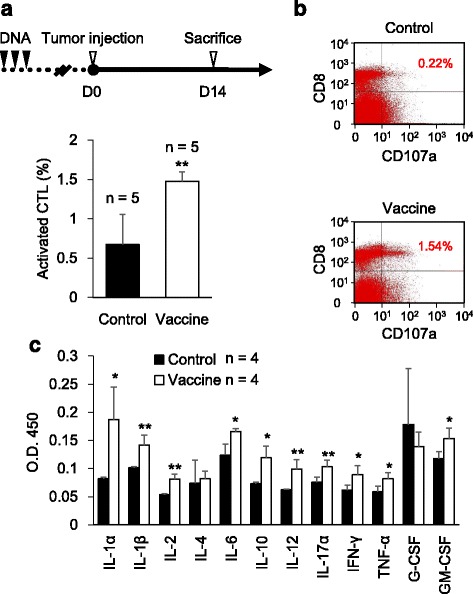
*LMP1*/pcDNA3.1 vaccination increases activated CTLs and induces proinflammatory cytokines in vivo. **a** *Upper panel*, schedule of vaccination and tumor injection. *Lower panel*, percentages (%) of CD8^+^/CD107a^+^ cells (activated CTLs) in mice from control or vaccine groups. **, *P* < 0.01. C57BL/6 J mice were vaccinated weekly with either empty pcDNA3.1 (control) or *LMP1*/pcDNA3.1 (vaccine) (*n* = 5) for 3 weeks. Subcutaneous injection of 1 × 10^6^ LMP1-expressing TC-1 cells was given 3 weeks after the last dose of vaccination (D0). Mice were sacrificed 2 weeks after tumor injection. Splenocytes were harvested and subjected for flow cytometric analysis of CD8 and CD107a expression. **b** Representative plots of flow cytometry from control or vaccine groups. Percentages of CD8^+^/CD107a^+^ cells (activated CTLs) are as indicated. **c** Statistical analysis of cytokine ELISArray. Sera from mice in control and vaccine groups (*n* = 4 in each group) were collected and subjected to ELISArray. Results are presented as mean ± S.D. *P < 0.05. **P < 0.01

To elucidate the mechanism of tumor suppression/prevention effect of *LMP1*/pcDNA3.1 vaccine, we examined the population of activated CTLs in vaccinated mice. C57BL/6 J mice were vaccinated with empty vector (control) or *LMP1*/pcDNA3.1 (vaccine) 3 weeks before subcutaneously injected with LMP1-expressing TC-1 cells and sacrificed 2 weeks after tumor injection (Fig. 5a, upper panel). Splenocytes were harvested for flow cytometric analysis. Results showed that the population of CD8^+^/CD107a^+^ cells (activated CTLs) was significantly larger in the vaccine group as compared with the control group (Fig. 5a, lower panel, *n* = 5, *p* < 0.01). Representative images of flow cytometry are shown in Fig. 5b. To analyze overall effect of LMP1 vaccine on circulating cytokines, we collected sera from the same animals and performed cytokine ELISArray. The results showed that significant elevation of proinflammatory cytokines, including IL-1α, IL-1β, IL-2, IL-6, IL-12, IL-17A, IFN-γ, TNF-α, and GM-CSF, was observed in mice in vaccine group as compared with mice in the control group (Fig. 5c, *n* = 4). These results suggest that *LMP1*/pcDNA3.1 vaccination significantly increases activated CTLs and induces proinflammatory cytokines in vivo.

### *LMP1*/pcDNA3.1 vaccination increases LMP1-specific T lymphocyte activities ex vivo

**Fig. 6:**
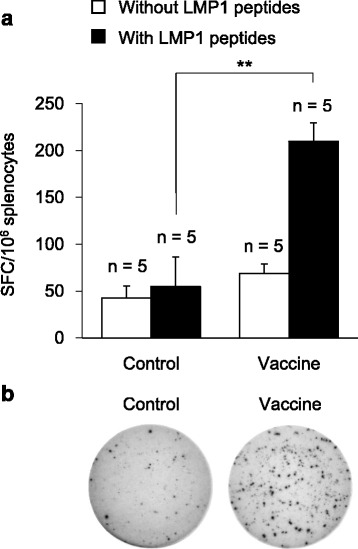
*LMP1*/pcDNA3.1 vaccination increases LMP1-specific T lymphocyte activities ex vivo. **a** INF-γ ELISPOT assay for evaluation of spot forming cells (SFCs) from control or vaccine groups with or without LMP1 activation. C57BL/6 J mice were vaccinated weekly with either empty vector (control) or *LMP1*/pcDNA3.1 (vaccine) (*n* = 5) for 3 weeks. Subcutaneous injection of 1 × 10^6^ LMP1-expressing TC-1 cells was given 3 weeks after the last dose of vaccination. Mice were sacrificed 2 weeks after tumor injection and splenocytes were harvested for ELISPOT assay. Splenocytes were incubated with or without LMP1 peptides for 24 h as indicated. **, *P* < 0.01. **b** Representative images of SFCs incubated with LMP1 peptides from control or vaccine group

To determine whether the activated CTLs in *LMP1*/pcDNA3.1 vaccinated mice were LMP1-specific, we performed ex vivo functional analysis using INF-γ ELISPOT assay. Results showed that, in the presence of LMP1 peptides, the number of spot forming cells (SFCs) in the vaccine group was significantly higher than in the control group, whereas no difference was observed between vaccine and control groups without LMP1 peptides stimulation (Fig. 6a, *n* = 5, *p* < 0.01). Representative images of SFCs from control or vaccine group are shown in Fig. 6b. These results indicate that *LMP1*/pcDNA3.1 vaccination successfully increases the population of LMP1-specific T lymphocyte.

## Discussion

In this study, we have established a LMP1-expressing TC-1 xenograft model in immunocompetent C57BL6/J mice. This tumor model allows researchers to evaluate the efficacy of various immunotherapies against EBV LMP1-expressing tumors such as NPC. Here, we demonstrate that *LMP1*/pcDNA3.1 vaccination significantly suppresses LMP1-expressing tumor growth and metastasis in vivo. We also show that LMP1 vaccine induces a panel of proinflammatory cytokines, including IL-1α, IL-1β, IL-2, IL-6, IL-12, IL-17A, IFN-γ, TNF-α, and GM-CSF. Furthermore, we demonstrate that LMP1 vaccine activates larger populations of LMP1-specific CTLs, indicating that LMP1 vaccine is a promising therapeutic modality.

EBV infects more than 90% of the population worldwide [18]. After primary infection during early adolescences, CTLs of the host controls, but does not eliminate, the virus [14]. During the carcinogenesis of NPC, EBV forms episomes in the epithelial cells and expresses restricted viral proteins such as EBNA1, LMP1 and LMP2 [3]. EBV develops several immune escape mechanisms that enable the survival of malignant cells harboring viral antigens. Tumor infiltrating lymphocytes in NPC secret IL-10, which can block CTL responses [19]. Immune recognition of EBNA1 is restricted by its internal glycine-alanine repeat that prevents proteasomal degradation and blocks endogenous presentation of CTL epitopes [11, 12]. Both LMP1 and LMP2 are able to avoid immune recognition [20, 21]. Despite these hurdles, both LMP1 and LMP2 have high expression in NPC and are relatively immunogenic [22, 23], rendering them suitable candidates for EBV-targeted immunotherapy. In this study, we demonstrate that LMP1-based DNA vaccine elicits LMP1-specific CTL activities and suppresses/prevents LMP1-expressing tumor development in murine models. This study proves our concept that LMP1 is a good candidate for targeting EBV-associated malignancies.

It is worth mentioning that both LMP1 and LMP2 have potentials to promote oncogenic transformation [13]. Consequently, the utility of vaccine encoding full-length antigens in patients is limited. Although none of the vaccinated animals in our study develop tumor on the vaccination site (observed till 3 months after vaccination, data not shown), modification of LMP1 vaccine by careful selection of CTL restricted epitopes will be the subject of future study in our laboratory.

Two types of therapeutic vaccines have been evaluated in clinical trials for NPC patients. The first type of vaccine is transfer of autologous dendritic cells (DCs) which are transfected with vectors containing EBV antigens ex vivo [24, 25]. The result of a phase II trial on LMP1/LMP2-based DC vaccine was not satisfactory because only two out of 16 NPC patients had partial response while the rest patients had stable or progressive disease [24]. Moreover, broad application of DC vaccine is difficult because of the high cost from generation of personalized DC. The second type of vaccine is injection of vectors encoding EBV antigens. In this category, LMP1 and LMP2 polyepitope vaccines were developed by Duraiswamy et al. and were shown to be effective in eliciting antigen specific CTL responses and suppressing tumor growth in murine models [7, 8]. The main difference between our study and previous studies is that we used LMP1-expressing TC-1 cells, a murine lung epithelial cell-line [26], as target cells instead of lymphoblasts. Being a pre-clinical study and in the attempt to mimic NPC, epithelial models is one step closer to resemble actual antigen processing machinery in human NPC. Currently, a phase I clinical trial with results of EBNA1/LMP2-based vaccine in NPC patients has been released and antigen specific CD4 and CD8 responses were reported [9]. Our study urges the need to incorporate LMP1 epitopes into EBV-targeted vaccine in order to achieve utmost treatment efficacies.

We show that a panel of proinflammatory cytokines are elevated by LMP1 vaccine treatment, including IL-1α, IL-1β, IL-2, IL-6, IL-12, IL-17A, IFN-γ, TNF-α, and GM-CSF. Among them, elevated IFN-γ is a strong evidence of induced cellular immunity and CTL activities. Interestingly, IL-10, which activates regulatory T cells and has anti-inflammatory effect, is also elevated in LMP1 vaccinated group. This result could be explained by previous report that LMP1 induces CD4 cells to secret IL-10 when processed and presented by dendritic cells [27].

Besides DNA vaccine, there are other EBV-targeted immunotherapies under development. The fact that EBV-specific CTL from peripheral blood of NPC patients can be reactivated ex vivo brings up a trend of adoptive autologous CTL therapy [6, 28]. Although adoptive CTL therapy in NPC gained effectiveness in phase II clinical trial [28], the specificity of CTLs activated by EBV-transformed lymphoblastoid cell lines (LCLs) are still under evaluation. The first concern is that EBV-transformed LCLs preferentially stimulate T cells specific for EBNA antigens rather than LMP1 and LMP2 [29]. Another concern is that the sustainability may vary by the extent of clonal expansion during ex vivo activation [30]. In this regard, combination with LMP1/LMP2-based DNA vaccine is another strategy to boost the expansion of adoptively transferred EBV-specific T cells in NPC patients.

## Conclusions

Conventional concurrent chemoradiation therapy for NPC achieves a high cure rate in localized disease [31]. However, patients with locally aggressive or metastatic disease continue to have a poor prognosis with a median overall survival time of 233 days despite multiagent treatment [32]. Association of NPC with EBV opens a door to the development of EBV-targeted immunotherapy. In this study, we proposed a novel LMP1 vaccine that augment LMP1-specific CTL activity towards LMP1 positive tumors. This treatment modality possesses great potentials as adjunctive therapy for patients with recurrent/metastatic NPC.

## Abbreviations

CTL: Cytotoxic T lymphocyte
EBV: Epstein-Barr virus
HD: Hodgkin’s disease
LCL: Lymphoblastoid cell line
LMP1: Latent membrane protein 1
NPC: Nasopharyngeal carcinoma
SFC: Spot forming cell
